# Three-week topical treatment with placenta-derived mesenchymal stem cells hydrogel in a patient with diabetic foot ulcer: A case report: Erratum

**DOI:** 10.1097/MD.0000000000014869

**Published:** 2019-03-08

In the article, “Three-week topical treatment with placenta-derived mesenchymal stem cells hydrogel in a patient with diabetic foot ulcer: A case report”,^[1]^ which appeared in Volume 96, Issue 51 of *Medicine*, “PDMSCs hydrogel (cell number: 1 × 10^6/cells/cm2)” should be written as “PDMSCs hydrogel (cell number: 1 × 10^6/cells/ml).”
